# Nighttime Cough Characteristics in Chronic Obstructive Pulmonary Disease Patients

**DOI:** 10.3390/s25020404

**Published:** 2025-01-11

**Authors:** Albertus C. den Brinker, Okke Ouweltjes, Ronald Rietman, Susannah Thackray-Nocera, Michael G. Crooks, Alyn H. Morice

**Affiliations:** 1 Independent Researcher, 5708 DJ Helmond, The Netherlands; 2 Philips Digital Standardization & Licensing Research, 5656 AE Eindhoven, The Netherlands; okke.ouweltjes@philips.com; 3 Philips I&S, Innovation Engineering, Data Science and AI, 5656 AE Eindhoven, The Netherlands; ronald.rietman@philips.com; 4 Department of Academic Respiratory Medicine, Centre for Cardiovascular and Metabolic Research, Hull York Medical School, Cottingham HU16 5JQ, UK; susannah.thackray-nocera@nhs.net (S.T.-N.); michael.crooks@nhs.net (M.G.C.); a.h.morice@hull.ac.uk (A.H.M.)

**Keywords:** COPD, cough, acute exacerbation, alert

## Abstract

Coughing is a symptom of many respiratory diseases. An increased amount of coughs may signal an (upcoming) health issue, while a decreasing amount of coughs may indicate an improved health status. The presence of a cough can be identified by a cough classifier. The cough density fluctuates considerably over the course of a day with a pattern that is highly subject-dependent. This paper provides a case study of cough patterns from Chronic Obstructive Pulmonary Disease (COPD) patients as determined by a stationary semi-automated cough monitor. It clearly demonstrates the variability of cough density over the observation time, its patient specificity and dependence on health status. Furthermore, an earlier established empirical finding of a linear relation between mean and standard deviation of a session’s cough count is validated. An alert mechanism incorporating these findings is described.

## 1. Introduction

Cough is an important symptom occurring in many respiratory diseases and is associated with exacerbations, lung function decline and risk of death [1,2,3]. Increased cough is common during the acute exacerbation of COPD (AE-COPD), and a prodrome of increasing symptoms including cough can be seen for up to 2 weeks before AE-COPD. Thus, questionnaires for monitoring the status of asthma (ACT: Asthma Control Test) and COPD (CAT: COPD Assessment Test) include an assessment on cough. Calverley et al. [4] considered questionnaire-reported symptoms like cough, breathlessness, chest tightness and nighttime wakening and found a mean increase in symptom score of around 1 unit (scale: 0–4) at the time of exacerbation. This change in magnitude was found at a population level; in an individual patient, it is unlikely to prove clinically useful.

The reasons as to why questionnaire symptoms do not perform as hoped are various. One reason is the coarse quantisation of the response. Increasing the level of detail (like a visual analogue scale: VAS) is uncommon as the required introspection will likely become an issue; thus, posing questions that are hard to answer will typically come at the cost of decreased adherence. The interpretation of the labels associated with the questions are usually left to the user and this results in subjectivity. Furthermore, the moment that a patient tends to report a significant increase in the burden of a symptom (1-point on the questionnaire scale) is also likely the moment to seek advice, meaning that no additional lead time would exist even if the questionnaire scale is apt.

Out of the questions from the CAT (regarding cough, sputum, chest tightness and nighttime awakening), cough is the symptom with the highest concordance with AE-COPD, independent of COPD severity [4]. Cough is also a symptom that can be detected in an automated way, as shown in many technical studies on prototype cough detection systems. For reviews, see [5,6]. An automated system would mitigate issues associated with subjectivity, introspection and reporting. Sensors that are typically used are sound pressure sensors and/or accelerometers attached to the chest. Various systems are currently available such as wearable recorders [7], wearable sensors [8,9,10] and bedside systems [11,12].

For patient comfort and adherence, an unobtrusive and hassle-free system is preferred. Since day- and nighttime coughing are correlated, it would suffice to measure either. Measuring nighttime coughing has several advantages. It can be performed completely hassle-free by a stationary system in the home of a patient. Also, the nighttime is a period of identical behaviour (sleeping) while the daytime is agenda-driven and may involve all kinds of activities inducing unexplained day-to-day variability or requiring extra channels to collect relevant contextual information. Finally, using a microphone as a sensor in the sleeping quarters of a patient yields an off-body system, and the nighttime is also usually the most quiet period of the day, making sensing and detection more accurate.

For these reasons, the development of an unobtrusive stationary off-body microphone-based monitoring system was pioneered and has been reported in [13,14,15]. It holds the promise of constituting an element in an exacerbation prediction tool (for severe patients using non-invasive ventilation, the ventilator itself may provide the relevant information for tracking a deteriorating health status [16]). Further validation of this system was undertaken in a double-blind clinical study. This paper addresses a part of the outcomes of the study. It focuses on aspects of the cough monitor and cough behaviour (patient-specific patterns) such as the variability of the cough density over the course of a monitoring session and the validation of an earlier reported empirical relation between mean and standard deviation in case of a stable chronic coughing patient.

The outline of this paper is standard. Firstly, the clinical study set-up is discussed, its devices are then detailed and the data processing is described. Section 3 describes the findings and is followed by a discussion (Section 4).

## 2. Methods and Materials

### 2.1. Data Collection

We conducted a prospective longitudinal double-blind study of continual cough monitoring in COPD patients. To provide a reasonable chance of detecting AE-COPD, participants were studied for 12 weeks using domiciliary cough monitoring and asked to complete daily questionnaires each morning. If no exacerbation occurred in the first 12 weeks, the participants were asked to continue for at most another 3 months.

This study is double blind in the sense that (i) cough data were analysed without any knowledge of the patient status or condition and (ii) moderate and severe COPD exacerbations were identified retrospectively without access to the cough monitor data. The primary aim of the trial was to validate a (causal) alert mechanism for exacerbations based on cough trend data [14].

The current paper addresses the secondary aims of the study: to better understand cough behaviour and any associated potential improvements of the alert mechanism. The main contributions of this paper are twofold. First, we demonstrate patient variability in nighttime cough patterns in the form of a case study and show its dependence on health status. Second, we validate the empirical relation between mean cough count and cough count variation [14]. Both have been performed using only the data of the cough monitor, i.e., without access to the medical data, questionnaire data and identified COPD exacerbations.

This study was reviewed and approved by the North East-York Research Ethics Committee (REC Ref.: 21/YH/0203), the United Kingdom Health Research Authority and the Internal Committee Biomedical Experiments of Philips Research. Informed consent was obtained from all participants involved in the study. The patient target was set at n=40 and 2 patients failed to finish the trial. One withdrew almost immediately and the other one moved houses, leaving equipment behind. The data collection process ran from August 2022 to June 2024. Several issues delayed a speedy progress, including COVID-19, political changes affecting equipment, and organisational changes at Philips.

### 2.2. Cough Monitor

**Figure 1:**
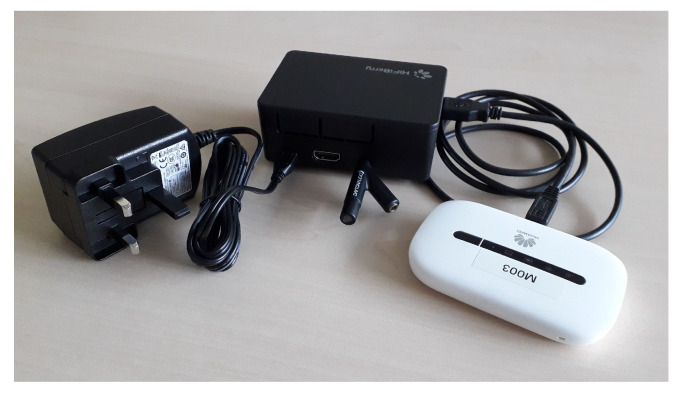
Bedside cough monitor: **left**: adapter; **mid**: single-board computer and microphone; and **right**: dongle.

The used cough monitor is a stationary system placed in the sleeping quarters of the participant. It is a successor to systems used in earlier studies [13,15], targeting the unobtrusive and hassle-free monitoring of cough. The prototype consisted of a single-board computer (ASUS Tinker Board 2G, ASUSTeK Computer Inc., Taipei, Taiwan) with a USB measurement microphone (Dayton IMM6, Dayton Audio, Springboro, Ohio) and a cellular dongle (Huawei E5330, Huawei, Shenzhen, China) (see Figure 1). Feature extraction and type of cough classifier were as in earlier trials [13,15]. The cough monitor was placed in the bedroom with a preference for the bedside table closest to the participant’s bed. No absolute control over positioning was possible as participant preferences and available power sockets influenced the decision.

To prevent issues with power outage, the system was created such that it would start up automatically. At the installation step, the system had a start and stop time that were set to 9 p.m. and 9 a.m., respectively. It was assumed that this covered the period of time that all patients spent in bed, and such a monitoring period is called a session. To generate timestamps and to start and stop monitoring at the correct moments, timing information was required. This information was drawn from the cloud. In view of the long period over which the monitor was intended to operate in a stand-alone mode, the system was shut down and booted every day, which is an effective means to prevent memory leakage. In case of failure to establish a connection to the cloud, the timing information is not available and the monitor will not start a session.

For the first 8 participants, the dongle contained an IoT SIM card (TruPhone) operating over a 2G network. Later participants used a 5G SIM card (Vodafone, UK). Data were transferred to the cloud (AWS) from where data were downloaded to a proprietary system for analysis. To ensure privacy, only timestamps and audio features were transmitted at moments where the soundscape changed. We refer to this as acoustic events or transients. For a limited number of these events, a one-second audio snippet was also recorded and transmitted. The limited use of short snippets prevented anyone from listening in to any conversation while enabling checking for audio issues with the devices or its set-up (e.g., a ticking clock next to the monitor can be detrimental) and creating a personalised classifier.

At the server side, the data were collected and a classifier was trained based on the first few days of monitoring, as described in [14]. In these previous trials, solid personalised classifiers were attained when trained with around 200 coughs. With such a target in mind, the number of days that require annotation (based on the snippet–feature combination) is variable because the number of acoustic events is highly dependent on the patient [17].

**Figure 2:**
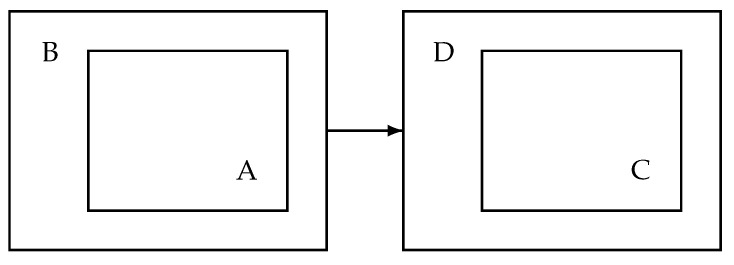
System overview: software components. (**A**) Audio processing in the patient’s home. (**B**) Scheduler and data transmission unit. (**C**) Cough classifier and alert mechanism. (**D**) Central data receiver and decryption unit.

A high-level schematic overview of the processing is given in Figure 2. On the left-hand side, the image shows the audio processing in the patient’s home. It consists of the audio processing based on an audio processing library denoted as A. The audio processing step captures the audio and extracts features and snippets. This part is a mature software component and has been deployed successfully in several trials. The audio processor is embedded in a scheduler with an interface and the communication part, including encryption. This has been developed for experimental purposes only. On the right-hand side, there is a data-receiving and development unit D, where data are received, decrypted and stored. Furthermore, there is an annotation and classifier training system to develop the personalised classifier based on the snippets and the associated subset of audio features.

**Figure 3:**
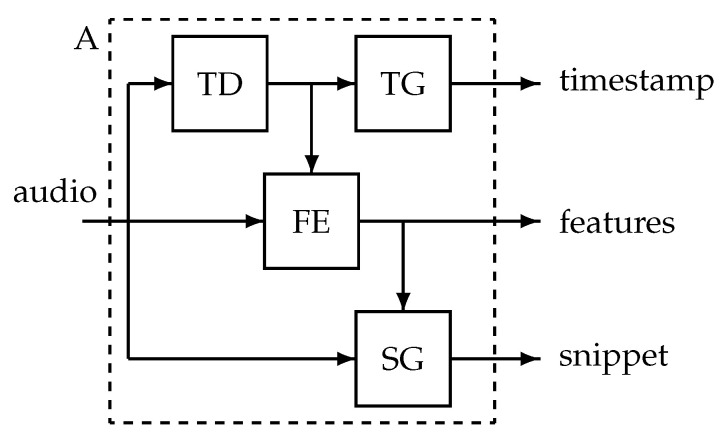
Data processing in the patient’s home. The audio data are input into a transition detector (TD). Transitions in the audio generate a timestamp in a timestamp generator (TG) and trigger the extraction of features in the feature extractor (FE). A snippet generator (SG) creates a sound snippet for a limited number of transitions.

**Figure 4:**
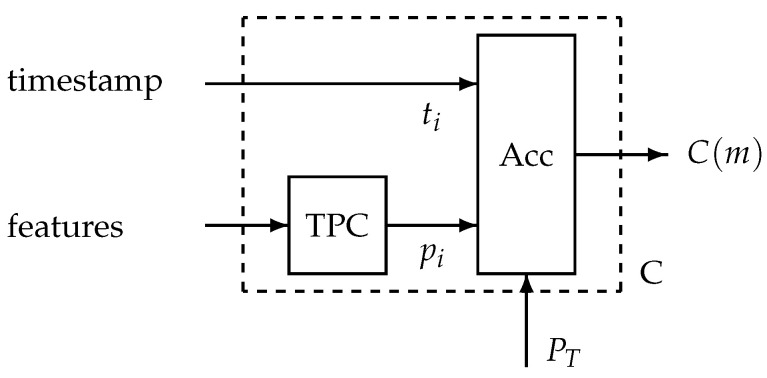
Data processing in system C. The features are input into a trained personalised classifier (TPC) generating the probability $p_{i}$. An accumulation unit (Acc) generates the number of coughs per session C(m) using the threshold probability $P_{T}$.

The data processing units A and C are further detailed. In Figure 3, the system A residing in the patient’s home is shown. A digital audio signal is the input, and system A creates a series of timestamps accompanied with sound features and, for a limited number of events, a sound snippet. The system contains a transition detector that triggers feature extraction and snippet generation. At the central side, the data processing unit C receives the timestamps and the features for each event *i* (see Figure 4). The trained personalised classifier creates a probability $p_{i}$ that the features stem from a cough sound. The timestamps $t_{i}$ and probabilities $p_{i}$ enter an accumulation unit. Here, events exceeding a threshold probability $P_{T}$ are counted over a monitoring session *m*, creating the session’s cough count C(m). This series is the input to the alert mechanism.

### 2.3. Cough Classifier

The cough monitor is closely tied to the operation of the human hearing system as this defines the ground-truth of what a cough is. This starts with the features that are extracted from the audio in the home of the patients. These incorporate a variant of Mel frequency cepstral coefficients (MFCCs). More precisely, band filters are used with equidistant spacing on an equivalent rectangular bandwidth (ERB) scale [18]. The filters are 3 ERB wide with 50% overlap. Next to these spectral features, energy levels before and after the acoustic event and the (local) density of events are extracted. The deployed classifier is a personalised classifier. A generic cough classifier would essentially require a one-class classifier, i.e., only using information on the sound of a cough without considering the environment in which the cough is being uttered. The neglect of using environmental information presumably leads to worse detection performance and is certainly not in line with our knowledge on human perceptual processing, which actively uses contextual information [19].

For annotation, an audio–visual interface was used. The audio is essential to annotate if the features belong to a cough, and not to an environmental sound or a vocal sound of the patient that is not a cough (like throat clearance, sigh, moan, sneeze, burp, speech, laughter). The signal waveform is provided, where a typical cough consists of three phases: an explosive part, an intermediate stage and a voiced phase. Not only is the visualisation helpful because of this specific pattern, but it is also instrumental to obtain a single identification of the cough over time and not multiple. The explosive phase of the cough is defined as the target as this is the acoustic response to the opening of the vocal chords after pressure build-up: a requirement for a cough by definition. It is also the most easily identified part of the cough: the intermediate and voiced phase are not always clearly present in sound and visuals. To obtain a unique signature for a cough, the position of the feature extraction is shown in the graph and the annotator checks if this corresponds to the explosive phase in case the sound resembles a cough.

For each patient, a classifier was trained using an extreme gradient boosted decision tree classifier (XGBoost 2.1.1 with binary:logistic classifier, python implementation) where the parameters [20] were left at their default values, except for max_depth =8, and num_boost_rounds =100, similar to what we had previously used in a general cough classifier that was trained on the data of many patients. We did not attempt to optimise each model’s performance by further tuning its parameters. After training the classifier, it is executed on the entire feature set using a relatively high threshold $P_{T}=0.9$. The outcome is a list of timestamps of detected coughs. To create a profile of the cough density over the monitoring periods, the following procedure was used. One-hour periods were selected and shifted over the monitoring period with a fifteen-minute update. The detected coughs in that period were counted. Data from the same time interval in all sessions were collected and treated as a random variable: mean, standard deviation, median and quartiles were calculated. Also, the α-trimmed mean was calculated with α=0.25.

Note that we prefer to use the term cough densities rather than cough frequencies. Frequency is a term connected to regularly occurring events, periodicity and quasi-stationarity. Since the number of coughs within a given timespan is far from periodic or equally spaced over time, the term cough density is preferred.

### 2.4. Alert Mechanism

**Figure 5:**
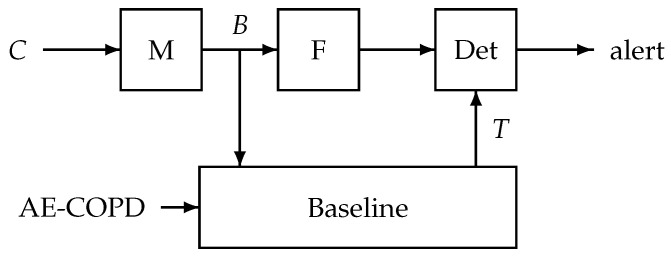
The original alert system where the cough counts *C* are mapped (M), smoothed (filter F) and fed to a detector unit (Det). The threshold level *T* in the detector is derived from a baseline search using mean filtered cough counts *B* excluding exacerbation days.

The alert mechanism defined in [14] is shown in Figure 5. The cough count C(m) is mapped to the B-scale by B(m)=αlog(1+βC(m)). Its output B(m) is smoothed using a first-order IIR filter and the smoothed signal is input to a detector where an alert is raised if this signal exceeds a threshold twice in three consecutive days. The threshold *T* is defined using a baseline search over the days, excluding exacerbation days (AE-COPD input), where the baseline is defined as the minimum of the means over the cough counts of nine consecutive days. This method is not useable in the present situation due to the double-blind character of this study nor is it compatible with a real-time system.

To solve this, an adaptive threshold was developed, which is also more appealing for long-term use in real life to allow for, e.g., ageing and seasonal effects. The method works as follows. The time series is mapped to the B-scale to be able to work with an identical outlier setting for all patients [14]. Data of the latest *L* sessions are input to determine a baseline. The oldest L−D sessions are used to define the current threshold; *D* is the delay or dead zone. Finally, the median is taken over the L−D observations and this median is input to a first-order IIR filter with pole $p_{t}$ and *z*-transform H(z) with

(1) $$H\left(z\right)=\frac{1-p_{t}}{1-p_{t}z^{-1}}.$$

An offset *O* is added to the output of the filter defining the dynamic threshold level T(m).

An alert is raised based on the smoothed cough counts on the B-scale using a first-order IIR filter with pole *p*. If this smoothed cough count exceeds the threshold *T* at least twice in the latest three sessions, an alert is raised. The settings for the mechanism are given in Table 1. We note that the parameter α, β, L−D and *p* correspond exactly to those in [14]. The parameters associated with the adaptive mechanism (*D*, $p_{t}$ and *O*) were set based on the cough data only, i.e., without knowledge of diagnosed exacerbations. For example, taking a small pole $p_{t}$ ($0<p_{t}<0.3$) gave less smoothing and alerts, which were considered as accidental. A large setting of the pole ($0.6<p_{t}<1$) gave increased smoothing with slow adaptation to decreasing cough counts. For simplicity, the pole was set to $p_{t}=0.5$ without extensive optimisation. Fine-tuning has to follow with the knowledge of the actual exacerbations and may even require more patient data.

**Figure 6:**
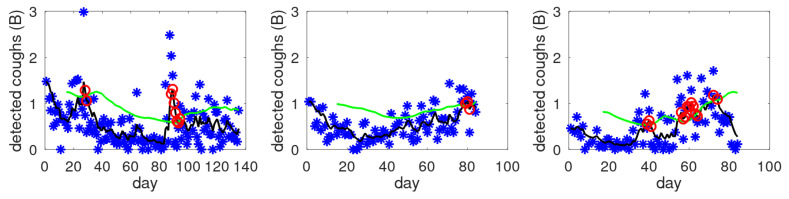
Examples of the cough count (blue asterisks), smoothed data (black line), dynamic threshold level (green line) and alerts (red circles).

To illustrate the behaviour of the alert mechanism, Figure 6 shows three examples of cough count (B-scale), smoothed cough count and constructed dynamic threshold *T*. All three examples show alert days as indicated by the red circles. The red circles are on the black smoothed line, as this is the input to the alert mechanism. Clinical aspects of the study and validation of this particular alert mechanism will be covered elsewhere. In the remainder, we will concentrate on the more general aspects of cough counting: cough density profiles and validation of the earlier proposed cough scale (B-scale used in Figure 6). The alert settings do not influence the later discussed cough counts and cough density profiles.

## 3. Results

### 3.1. Patient Characteristics

From the forty enrolled patients, one withdrew and another did not finish the trial. The characteristics of the remaining 38 patients are given in Table 2. Compared to our earlier studies [13,15], less-severe patients were involved in this trial.

Not all patients provided useable cough data. This is a consequence of the set-up of the study (retrospective without interventional mechanism), the behaviour of the patients (absence (e.g., holidays), moving) and any system issues (disconnected power, interrupted data connection). From the 38 patients, 4 were immediately excluded from further analysis. There were three patients (P035, P038 and P039) with severe connectivity issues, and patient P012 had connectivity and RF interference issues. It made the data too scarce to run the alert mechanism and, in most cases, there were even too few data to train a classifier.

**Figure 7:**
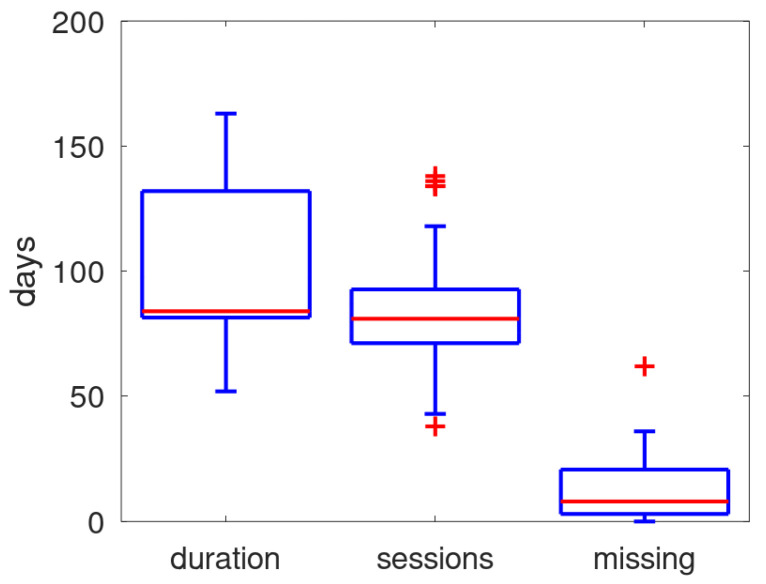
Boxplot of duration, sessions and unmonitored days. The red line identifies the median, the blue box gives the quartile range, the whiskers provide the full range except for outliers (red crosses).

In Figure 7, the distribution of the duration of the monitoring (number of days from first to last received data), the number of days within the period that data were received and the number of missing days (i.e., the difference between duration and monitoring sessions) is shown in a boxplot. The median of missing days is about 10, on a median of 84 days. Although the patient could have turned off the power, it is presumed that the main cause for missing days is that the system was unable to schedule its daily monitoring session due to an unguaranteed mobile connection.

### 3.2. Annotation and Classifier Performance

For each patient, a trained classifier was created by annotating the snippets that were transmitted. During annotation, it was found that the monitor had issues with RF interference, which appeared in the audio as spike trains. Next to patient P012 (already ruled out because of connectivity issues), this interference made the audio snippets (and audio features) for P020 questionable. With other patients, RF interference occurred much more rarely and is expected to have no or little impact on the results.

It was also observed that for one patient (P018), the dominant respiratory acoustic event was not a cough but what sounded like a combination of a cough with a throat clearance. The sound and waveform patterns observed in annotation thus did not match this case as the annotator was instructed to identify the explosive part in a normal three-stage acoustic event exhibiting intermediate and voiced phases after the explosive phase. No attempt was made to define an annotation process suited to this singular case. The data of this patient were excluded from further analysis. For more details on the annotation, see Appendix A.

Setting P018 and P020 aside, the number of patients with cough data became 32. From these patients, 12 had partners each and 20 did not. From the twelve partners, four were reported as coughers, four were not, and for the other four cases, this is unknown.

The experience from earlier experiments indicated that solid classifiers were obtained when around 200 coughs were used in the training. The number of snippets that were annotated as coughs ranged from 178 to 278 over the patients. The number of annotated non-coughs ranged from 256 to 8779, clearly marking the huge range of cough prevalence in the annotated snippet set.

Another observation that was made during data screening and annotation was that it is questionable whether patient P027 was actually occupying the sleeping quarters; after an initial period of about 8 weeks, both acoustic events and coughs dropped dramatically for almost all days. With other patients, there are also questions around presence as there were days with abnormally low numbers of acoustic events and coughs. No mechanism was constructed to rule out these days.

**Figure 8:**
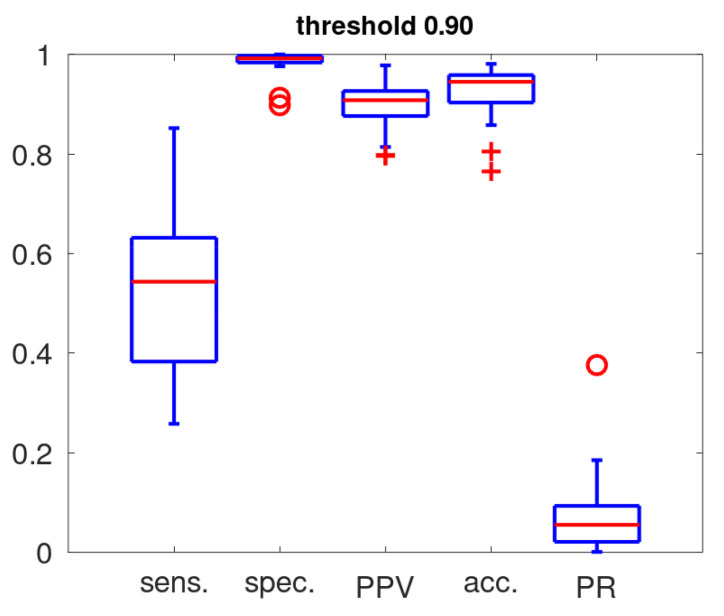
Boxplot of performance metrics of the cough classifier (sens.: sensivity; spec.: specificity; PPV: positive predicted value; acc: accuracy) and rate of the detected coughs (PR: positive rate) for the 32 patients. The red line identifies the median, the blue box gives the quartile range, the whiskers provide the full range except for outliers (red crosses) and extreme outliers (red circles).

Figure 8 gives the boxplots representing the sensitivity, specificity, accuracy and positive predictive value as determined in the training with a threshold setting of $P_{T}=0.9$ for cough classification. The fraction of acoustic events classified as coughs (positive rate: PR) on the entire data set is also included. These statistics highlight our basic notions on the design of an alert mechanism: a high PPV is essential if the cough counts are to act as its substrate. In view of the low cough prevalence [17] and robustness for exposure to untrained acoustic events, a high threshold ($P_{T}=0.9$) was set for the cough classifier. This ensures a high PPV by favouring specificity over sensitivity. The sensitivity ranged from 0.26 to 0.85, the specificity was high (all except two above 0.975), the full PPV range stretched from 0.8 to 0.98 and the accuracy was in the range from 0.86 to 0.98 (except for two patients).

**Figure 9:**
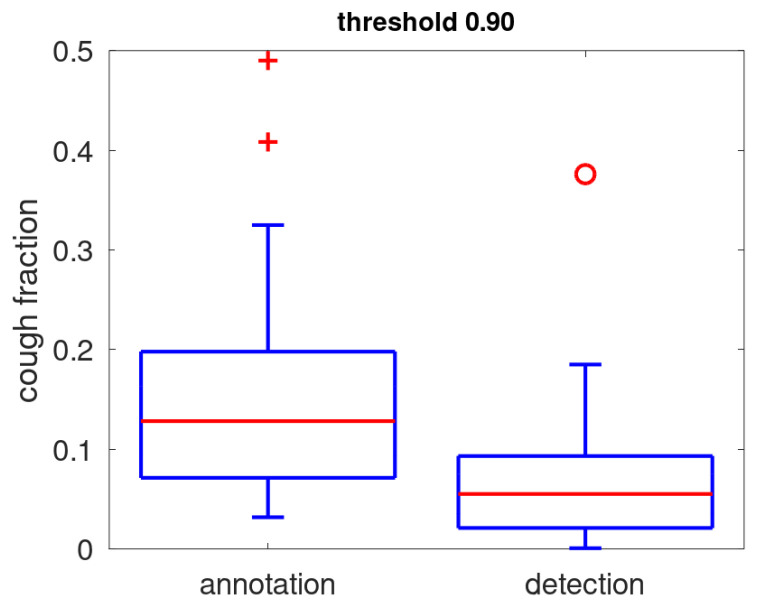
Boxplot of the prevalence of coughs as determined during annotation and positive rate (fraction of acoustic events classified as coughs for a threshold setting of 0.9). The red line identifies the median, the blue box gives the quartile range, the whiskers provide the full range except for outliers (red crosses) and extreme outliers (red circles).

In Figure 9, the prevalence of coughs as determined during annotation is compared to the positive rate. We note that the positive rate (detected coughs) is low for 75% of the patients below 10% of the acoustic events. There is one clear outlier where a quiet acoustic environment is combined with a large amount of coughs, resulting in a positive rate of nearly 0.4. The positive rate is smaller than the annotation prevalence; the median is 6% while the median cough annotation prevalence is 13%. This is mostly due to a low sensitivity induced by the preferred high threshold setting (median: 0.54). Other factors that contribute to the difference include the fact that prevalence, which is determined from annotation, covers only a small part of the full data, while the positive rate is determined over all acoustic events, and that the snippet selection is biased slightly towards the louder events in order to facilitate annotation.

### 3.3. Cough Counts

Each detected cough carries a timestamp. This enables statistics of these cough counts where we consider the mean and variance of the counts over a monitor session and cough density profiles over the monitoring period (9 p.m. to 9 a.m.). The amount of coughing depends heavily on the participant.

For each patient, it was checked if an alert was raised by our prototype alert mechanism, in which case the cough count would be unstable over the days. As the start of the alert track is delayed because the system needs time to collect baseline characteristics, the trend plots may reveal considerable variation in the beginning without an alert being generated. Therefore, a visual inspection of the data was undertaken (by the first author) to verify if the first part of the cough data did not have an upward or downward trend. In case of an absence of alert and absence of trend, the patient cough data are labelled as stable. An exception to this rule was P021 showing an enormous spread in cough counts in the middle of the observation period, and this was labelled as unstable as well. Only 6 out of the 32 cough trends were labelled as stable, and about half (17 out of 32) had at least 1 alert. An overview of this data is displayed in Appendix B.

**Figure 10:**
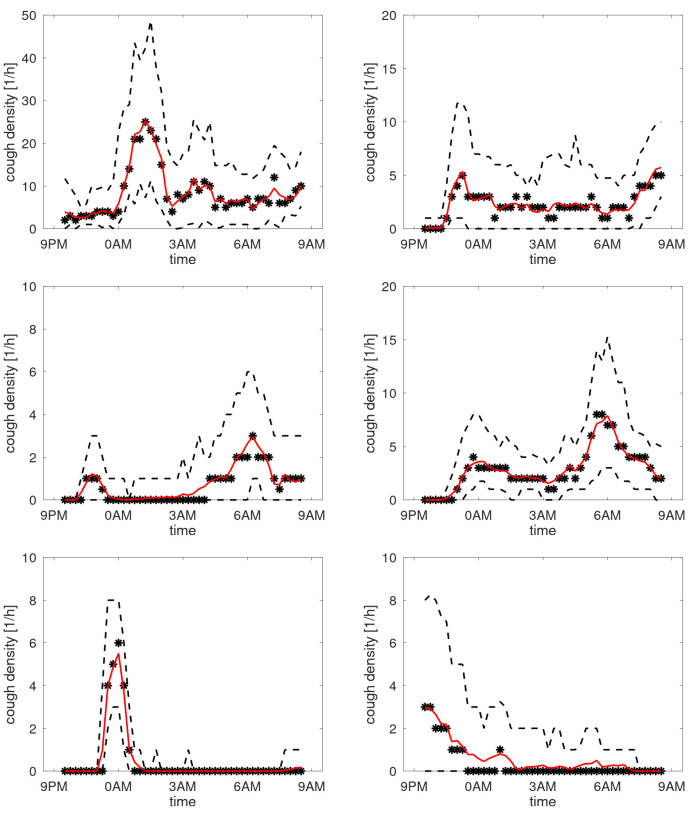
Cough density profiles over the monitoring period (9 p.m. to 9 a.m.) for participants with stable cough counts over the monitoring sessions. Black asterisk: median count; red line: trimmed mean; and dashed lines: quartiles.

In case of stable cough counts over the days, the averaging over the day creates a cough density profile presumed to be characteristic for that particular patient during normal respiratory condition. Figure 10 shows the profiles of participants with a stable cough count. In the cough density plots, the median density profiles are provided (expressed in coughs per hour) together with the quartile ranges and the α-trimmed mean with α=0.25. We observe that these curves are not uniform but exhibit peaks at different time instances. Some graphs have peaks in the morning hours, while some in the evening, with the latter presumably corresponding to the time the patient goes to sleep. Some patients hardly cough at all in the sleeping hours, yet some have a steady plateau in their cough density plots. The diversity of patterns agrees well with the observations in [14]. For completeness, the set of all cough density patterns is provided in Appendix B.

**Figure 11:**
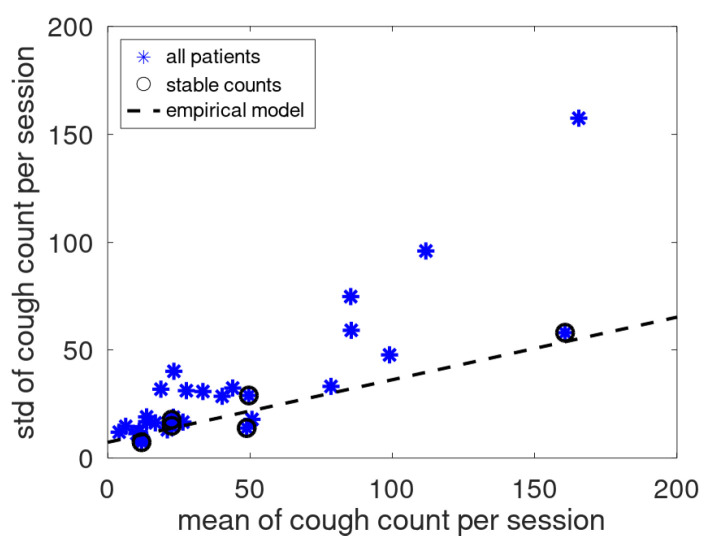
Standard deviation versus mean of the cough count per patient. Each asterisk represents a trial participant. Data from patients with stable cough counts are indicated by a black circle surrounding the asterisk. The dotted line represents the empirical relation from [14].

In [14], a linear relation was found between mean and standard deviation of cough counts of patients that had no medical issues during the monitoring period. To validate this empirical relation for the present cough data, the current data and the earlier found model are jointly plotted in Figure 11. The data of the assumed stable patients follow the earlier reported linear trend, while the data of the other patients are located dominantly above this line.

**Figure 12:**
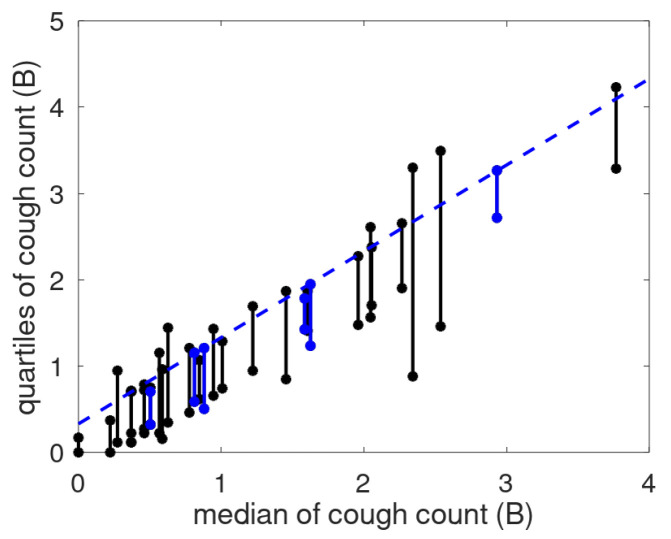
Quartile range as a function of the median cough count on the B-scale. Each bar represents the Q1–Q3 range for a patient. The patients with a stable cough count are represented by blue bars, else a black line is used. The dashed blue line indicates the median shifted vertically by 0.33 B.

The linear relationship suggested a scale transformation into a cough count scale (called the B-scale), where a unit step has the same meaning anywhere on the scale [14]. To show the effect of the scale, we provide the cough count quartile ranges as a function of the median in Figure 12. The dashed line, representing the median increased by fixed offset, runs congruent with the third quartile, especially for the patients with a stable cough count. For the non-stable patients, the bars occasionally extend above the line, and sometimes agree with it. This is assumed to be caused by the use of robust statistics, implying that if the data are mostly stable, the used metrics will follow that of the stable patients. Note that there were two patients (P017 and P027) with low median cough count and bars below the blue line. This may have been caused by a prolonged absence of the patient.

**Figure 13:**
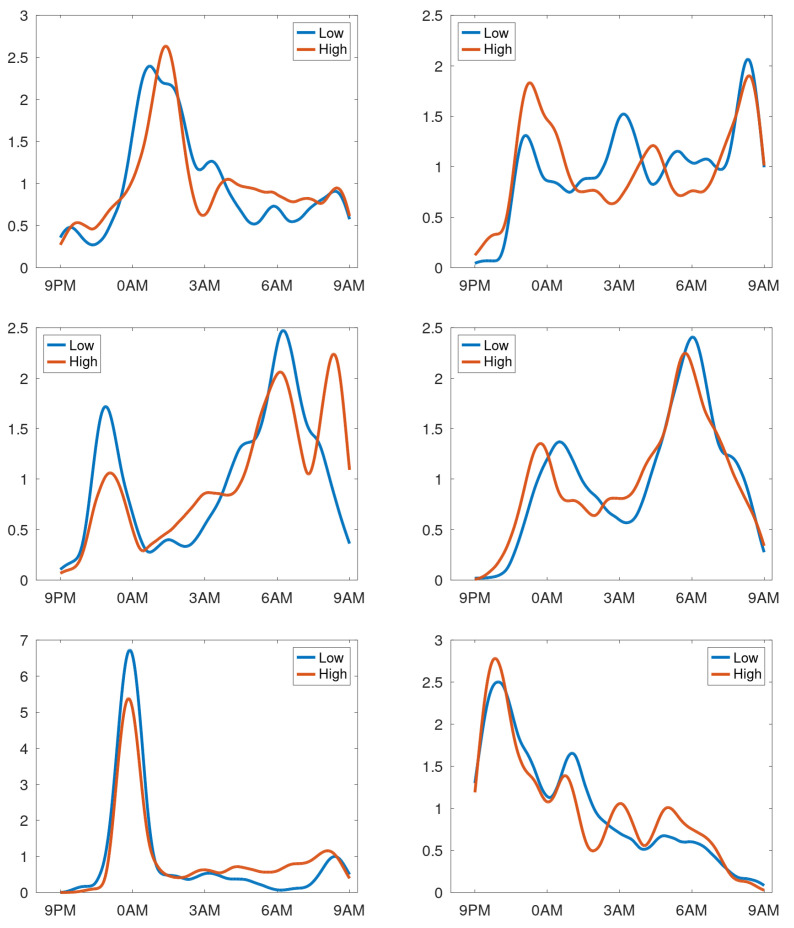
Normalised cough density profiles over the monitoring period (9 p.m. to 9 a.m.) for participants with stable cough count with data split according to sessions with low and high cough counts.

**Figure 14:**
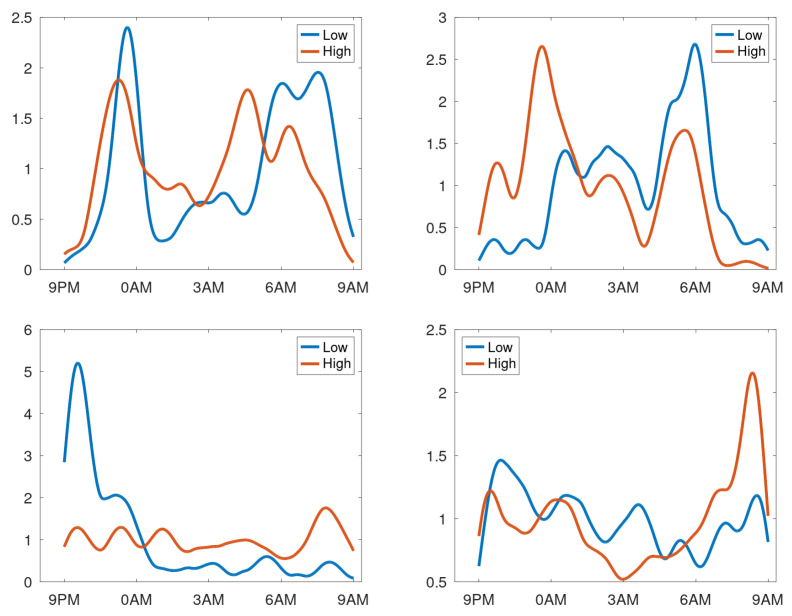
Normalised cough density profiles over the monitoring period (9 p.m. to 9 a.m.) with data split according to sessions with low and high cough counts. Four examples from patients having an unstable cough count over the trial period are shown.

As previously mentioned, creating profiles by averaging over days for patients having respiratory issues during the observation period may not provide a meaningful density profile. To test this hypothesis, the data of each patient were split into two equal parts: sessions with the highest and lowest counts. Profiles were created for these two data sets. To compare these profiles visually, they were normalised such that the mean density equalled 1 cough/h. In Figure 13, the profiles are shown for patients having stable cough counts. Our interpretation is that the density profile of a stable patient is only slightly dependent on the overall session cough count. In contrast, four profiles of patients with an unstable cough trend were selected and are shown in Figure 14. Here, we observe that clear shifts in the densities occur. The top-right graph reveals that for days with a high cough count, an additional peak occurs around midnight. The bottom-right shows a heightened peak in the morning hours. The bottom-left completely changes its character from coughing dominantly in the evening (at around 10 p.m.) to coughing all night long. The top-left graph has a change in timing; the evening peak occurs earlier and shows an even clearer shift in the two peaks in the morning. This would be compatible with a patient going to bed earlier and sleeping for less hours in case of increased cough count and thus a presumed respiratory issue.

## 4. Discussion

For COPD patients with an appreciable nighttime cough count, a cough density profile can be constructed. These density functions tend to be rather uniform (flat), have one peak (morning or evening cough) or are double-peaked (morning and evening cough). The results confirm the patterns shown in an earlier study [14] and demonstrate a large variation over the population.

The deployed method of creating density profiles uses averaging over all days. To prevent bias, we considered only patients with data where the alert mechanism was not triggered and visual inspection did not contradict the assumption of similar behaviour at different days. Results from all other patients are presented in Appendix B. Even though robust statistical measures were used, they may be biased and therefore should preferably not be used as a description of the patient’s normal cough density. Nevertheless, these curves also show pronounced patient-specific peaks.

Note that for some patients, the density profile is relatively large or even maximum at or near the start or stop of the monitoring period. This is an undesired monitoring setting as it means that the count can be largely influenced by slight changes in habits of the patient. It may be associated with acoustically open spaces or deviant sleeping hours.

For stable patients, an empirical relation has been proposed between mean and standard deviation of the nighttime cough count [14] (for unstable patients, this is not meaningful; mean and standard deviation estimates are rather sensitive to outliers). This relation was verified by considering data from cough counts where the cough count trends were identified as stable.

The earlier introduced B-scale for cough count was applied to the present data. It confirms that this scale enables defining a patient-independent offset which, when added to a robust statistic for the baseline level, defines a cut-off for identifying outliers. In [21,22], the relation between standard deviation and mean was also reported. In the paper, the slope was found in the range from 0.37 to 0.40, while here, we consider 0.29. The main causes attributing to this difference are assumed to have been the following:

- Our approach considers only data from stable epochs, obviously leading to a lower slope; see Figure 11. The choice for stable epochs is relevant in view of the non-robust nature of mean and standard deviation statistics.
- The monitor is tuned to a high specificity as a consequence of personalisation and using a high classification threshold. This means that contributions of environmental sound variability (seeping in via false positives) are suppressed to a high degree.
- The considered data are restricted to COPD patients and nighttime monitoring.

We considered the dependence of the cough count per night on the density. This confirmed that the shapes of the density profiles were only slightly dependent on the strength for stable patients. For unstable patients, the shape of the density profiles may vary considerably with the total amount of coughs per night, and thus presumably with the patient’s respiratory health.

We note that the reported findings on profiles are restricted to COPD patients. We also note that the proposed monitoring system differs from other cough monitors (e.g., [8,9,10,11,12,21]) in that it is not only capable of providing information on the amount of coughs to the user or caregiver but has the additional functionality of an alert mechanism for AE-COPD. Further explorations with this system should mitigate the observed RF interference, include a more stable mechanism for connectivity, and address the fine-tuning of parameters.

## 5. Conclusions

We analysed data from COPD patients (N=32) obtained by a stationary nighttime cough monitor. Cough density profiles were created, demonstrating a large degree of variation in cough behaviour across patients. The empirical relation between mean and standard deviation of the nighttime cough count was confirmed. The effect of mapping the cough scale for uniform outlier detection was illustrated. These findings deepen our insights and may serve as guidelines in the design for clinical applications like alert mechanisms for chronic coughers. The data also suggest that not only the intensity (amount of coughs in a day or night) but also the shape of cough density profiles may vary substantially with the patient’s respiratory state. Validation of the alert mechanism incorporating these insights will be our next step.

## Figures and Tables

**Table 1:** Alert mechanism settings. Parameters for mapping, smoothing and creating a dynamic threshold level.

	Parameter	Setting	Units
Mapping to B-scale	α	3.45	B
	β	0.04	
Smoothing of data	*p*	0.75	
Baseline creation	*L*	14	days
	*D*	5	days
	$p_{t}$	0.5	
	*O*	0.35	B

**Table 2:** Baseline demographics for study participants. Values are expressed as median and range (in brackets). BMI: Body mass index; FEV1: forced expiratory volume in 1 s; CAT: COPD assessment test; VAS: visual analogue scale for cough; and HARQ: Hull airway reflux questionnaire. Not all data reflect the full cohort indicated by *, N=33, and ^*o*^, N=13.

Characteristic	N=38
Gender: Male/Female	24/14
Age (years)	72 [57–84]
Weight (kg)	79 [44–173]
Height (cm)	168 [152–198]
BMI (kg/m^2^)	27.7 [16.2–41.3]
Smoking status	
Current/ex	7/31
Pack years	46 [10.5–212]
FEV1 (L)	1.13 [0.61–2.81]
% predicted FEV1	43 [20–106]
CAT score	
begin	27 [5–37]
end *	25 [12–36]
VAS	30 [0.5–85]
HARQ	40 [8–70 ]
Exacerbations ^*o*^ (1/yr)	3 [1–7]
Admissions ^*o*^ (1/yr)	0 [0–2]

**Figure A1:**
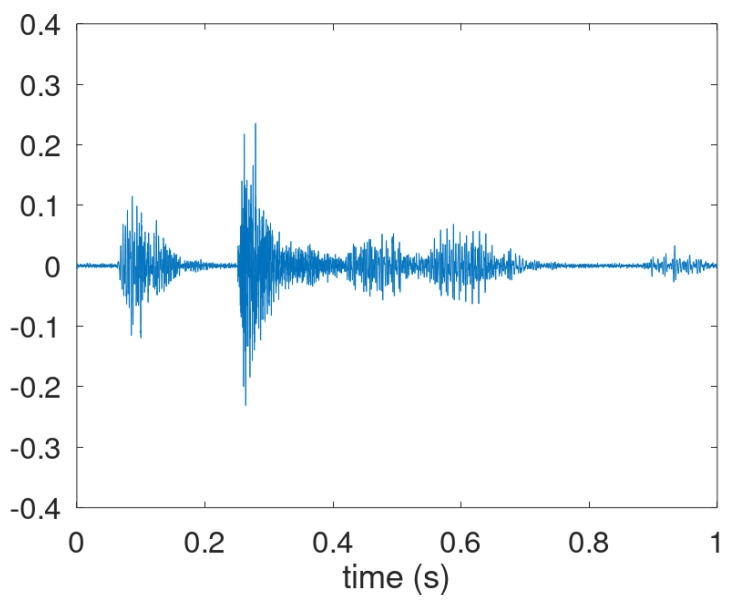
Cough sound waveform with recognizable 3-phase motor act.

**Figure A2:**
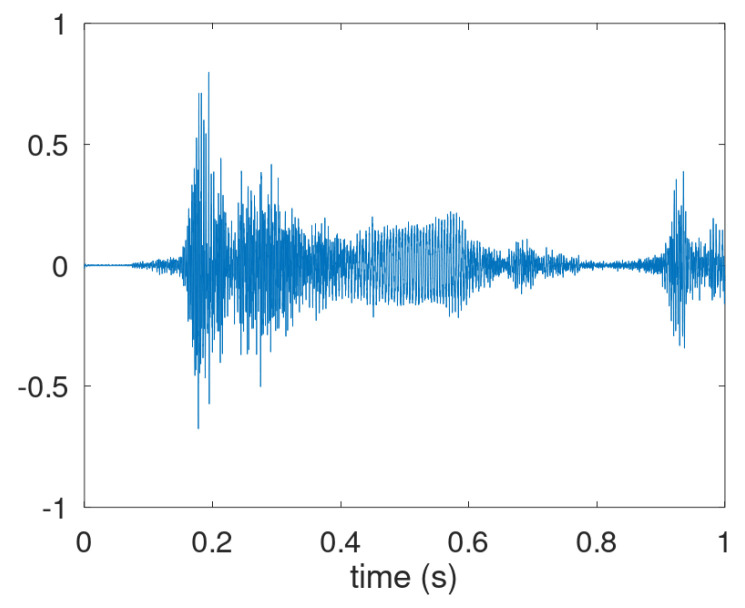
Prototypical waveform of a cough consisting of three distinctive parts in the airflow expulsion.

**Figure A3:**
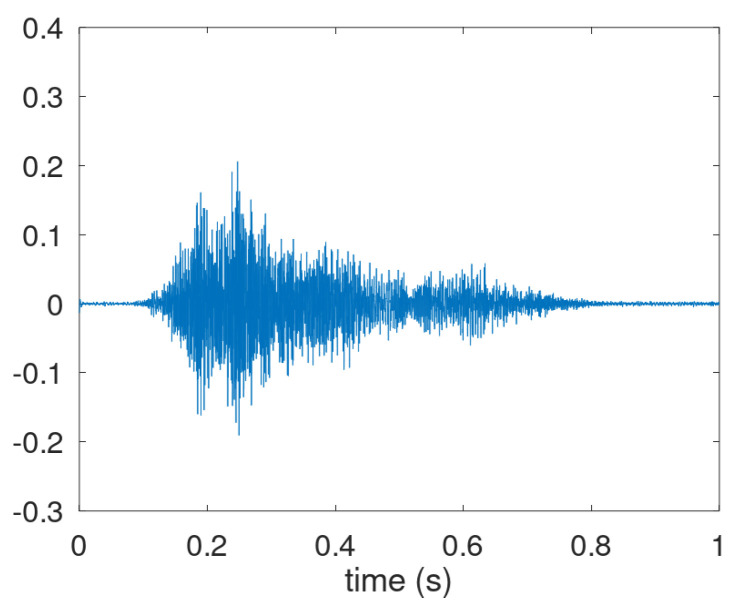
Prototypical waveform of a cough sound where the voiced phase in the airflow expulsion appears absent.

**Figure A4:**
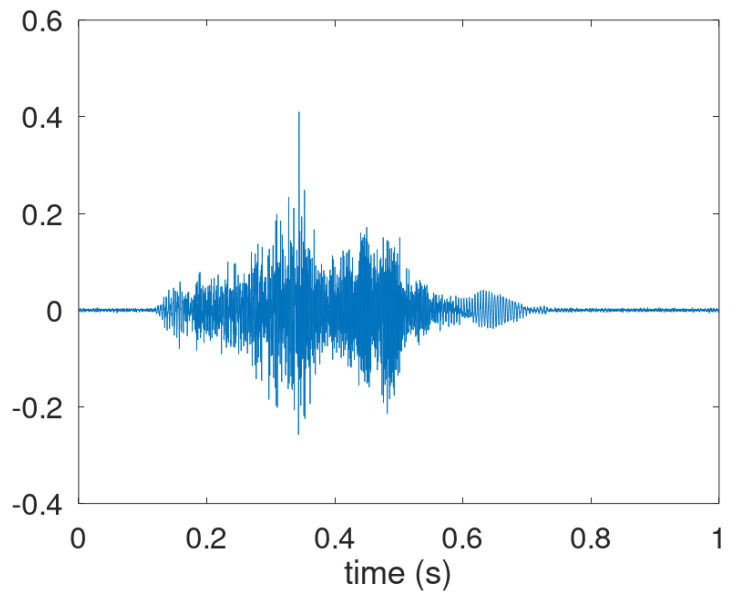
Dominant waveform of acoustic adverse respiratory events for P018.

**Figure A5:**
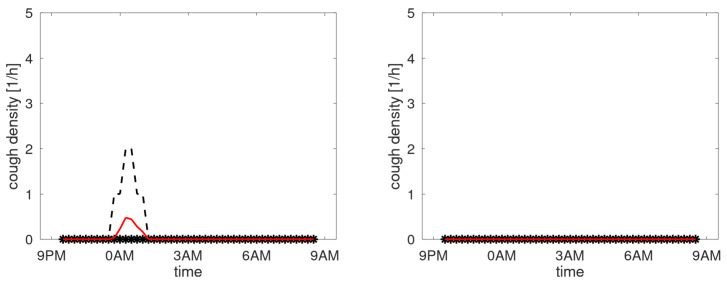
Cough density profiles over the monitoring period (9 p.m. to 9 a.m.). Participants with the lowest density. Black asterisk: median count; red line: trimmed mean; and dashed lines: quartiles.

**Figure A6:**
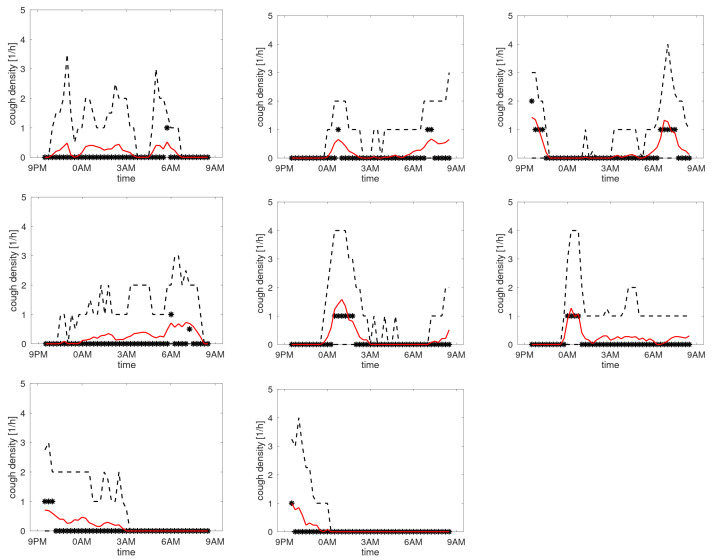
Cough density profiles over the monitoring period (9 p.m. to 9 a.m.). Participants with low density. Black asterisk: median count; red line: trimmed mean; and dashed lines: quartiles.

**Figure A7:**
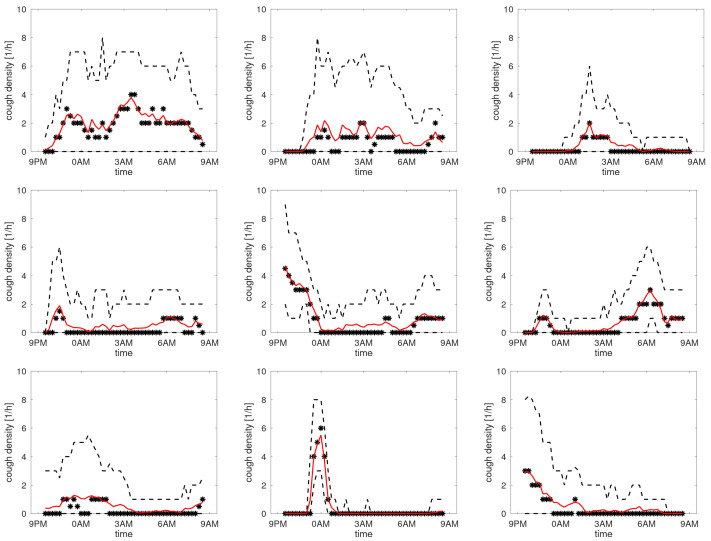
Cough density profiles over the monitoring period (9 p.m. to 9 a.m.). Participants with medium density. Black asterisk: median count; red line: trimmed mean; and dashed lines: quartiles.

**Figure A8:**
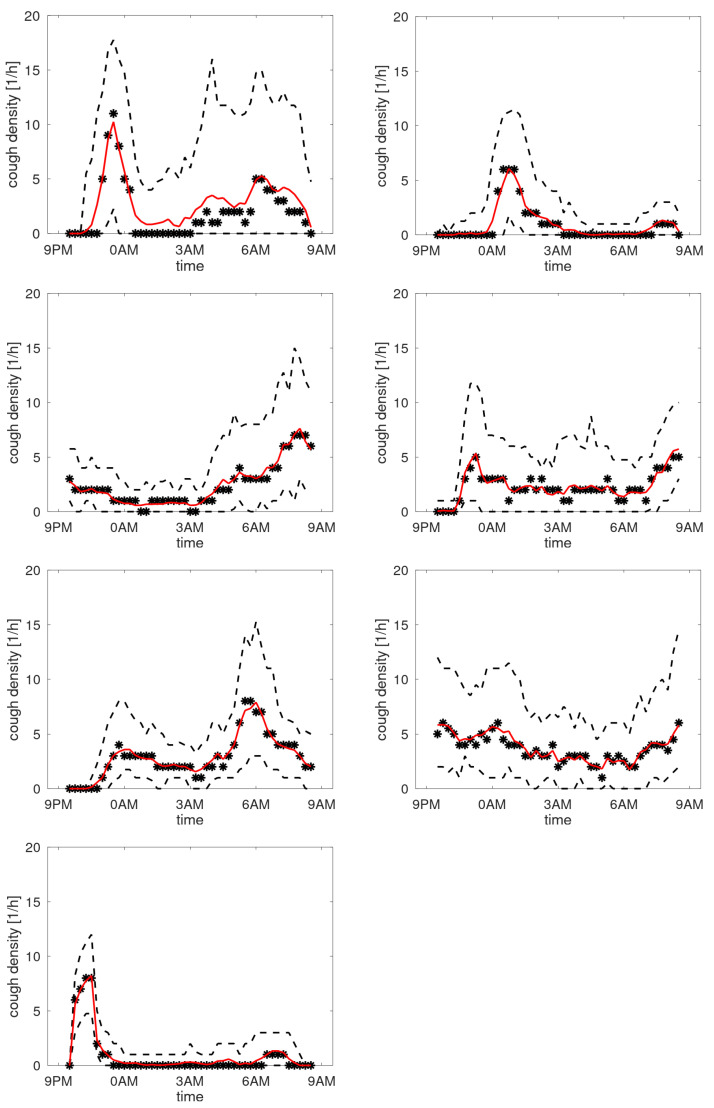
Cough density profiles over the monitoring period (9 p.m. to 9 a.m.). Participants with high density. Black asterisk: median count; red line: trimmed mean; and dashed lines: quartiles.

**Figure A9:**
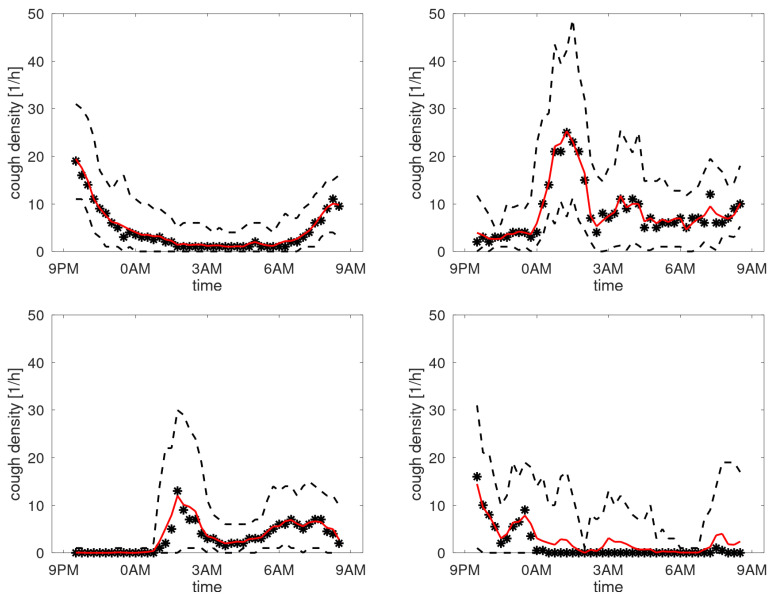
Cough density profiles over the monitoring period (9 p.m. to 9 a.m.). Participants with very high density. Black asterisk: median count; red line: trimmed mean; and dashed lines: quartiles.

**Figure A10:**
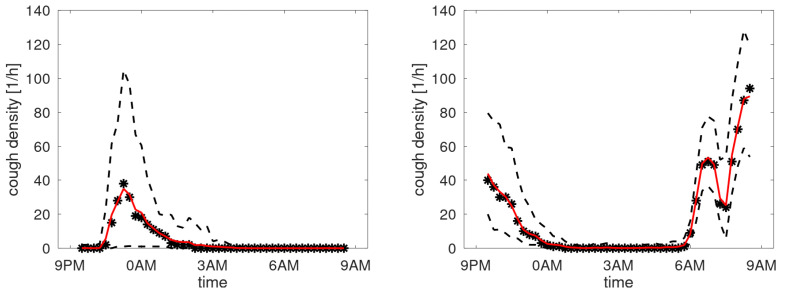
Cough density profiles over the monitoring period (9 p.m. to 9 a.m.). Participants with the highest density. Black asterisk: median count; red line: trimmed mean; and dashed lines: quartiles.

## Data Availability

Data collected in the clinical trial reside at Hull York Medical School.

## Appendix A. Cough Waveforms

In this appendix, we consider in more detail the annotation of cough and the usage of the cough waveform. Cough should basically be considered as a three-phase motor act featuring inhalation, pressure build-up and an expiratory airflow. An example of a sound waveform that illustrates this is given in Figure A1. It shows the inhalation effort (interval 0.05–0.15 s), the pressure build-up (0.15–0.25 s) and the expiratory airflow (0.25–0.75 s).

However, in many audio recordings, the inhalation phase is not visible nor audible. In the waveform of the expiratory motor phase, three distinctive parts are often discernible and they are defined as the explosive part, the intermediate part and the voiced part. In Figure A2, these parts are shown in the intervals 0.15–0.25, 0.25–0.40 and 0.40–0.60 s, respectively. We note that these three parts are also recognizable in Figure A1, though the transition from intermediate to voiced is harder to pinpoint here. We also note that the cough shown in Figure A2 is actually the start of cough bout; a second explosive phase is visible around 0.95 s.

The third phase is not always present, as shown in Figure A3. Here, the explosive part is followed by a decaying intermediate phase, but the voiced phase is not (clearly) visible in the waveform. Coughs having only an explosive part and a very short intermediate phase occur as well. Therefore, the annotation focused on audible coughs with a clear explosive part, i.e., associated with a release after the pressure build-up.

For Patient P018, cough waveforms like those illustrated in the figures so far were scarce. Instead, the dominant pattern of audible cough-like respiratory activity is shown in Figure A4. It combines throat clearance starting at 0.15 s with a cough. Possibly, the explosive part occurred at 0.35 s but it is integrated in the rising clearing sound. The intermediate and voiced parts are visible at 0.4 to 0.6 s and 0.6 to 0.7 s.

## Appendix B. Cough Density Plots

In this appendix, data are provided, underlying the validation of the B-scale, and cough density profiles of all patients are presented.

Table A1 gives the basic data concerning B-scale validation for all 32 patients, and the figure number containing the profile. The first column gives the patient ID and the second one shows the number of monitoring sessions. For B-scale validation, mean and standard deviation were measured and are shown in the last two columns. Furthermore, there are two columns concerning stationarity. The column Alert identifies if the alert mechanism flagged an exacerbation. The column Stable identifies if the cough data are stationary; it is said to be stationary if no alert was raised and a visual inspection of the cough graphs confirmed this. This visual inspection was undertaken since the alert mechanism is not functional in the initial period and downward trends may exist, making the cough count non-stationary but without raising an alert. In case of the absence of alert and the absence of a trend, the patient cough data are labelled as stable. An exception to this rule was P021 showing an enormous spread in cough counts in the middle of the observation period and this was labelled as unstable as well. Only 6 out of the 32 cough trends were labelled as stable, and about half (17 out of 32) had at least 1 alert.

Figure A5, Figure A6, Figure A7, Figure A8, Figure A9 and Figure A10 contain the cough density profile for all patients. These are organised in six plots with increasing cough severity. Table A1 provides the information of the association between patient and plot. In the cough density plots, the median density profiles (expressed in coughs per hour) are provided together with the quartile ranges and the α-trimmed mean. Figure A5 shows the profiles of participants with a third quartile staying below 1 cough/h. Essentially, these are not chronic coughers.

Figure A6 shows the profiles of participants with the maximum of the third quartile between 1 and 4 coughs/h. The median indicates that three participants tend to cough more around 0 a.m. (when going to bed) with one of these three also having an increased count in the morning.

Figure A7 shows the profiles of participants with the maximum of the third quartile between 4 and 10 coughs/h. Here, the picture is rather diverse, with participants having higher counts in the morning or evening, or a more uniform distribution over the sleep period.

**Table A1:** Specification of number of sessions, figure containing density plot, presence of alert, stability of cough trend, mean (μ) and standard deviation (σ) of the session cough count per patient. Processed data based on classifier probability threshold $P_{T}=0.90$. Y and N indicate Yes and No, respectively.

Patient	Sessions	Figure	Alert	Stable	Mean	Spread
					μ	σ
001	83	Figure A8	Y	N	85.6	59.2
002	48	Figure A6	N	N	23.3	40.2
003	78	Figure A9	Y	N	99.0	47.8
004	86	Figure A7	Y	N	43.9	32.5
006	61	Figure A8	N	N	33.5	30.8
007	76	Figure A7	N	N	40.2	28.6
008	50	Figure A7	N	N	16.7	16.3
009	133	Figure A6	Y	N	13.7	19.1
010	134	Figure A7	Y	N	23.1	18.9
011	81	Figure A6	Y	N	12.0	8.8
014	79	Figure A8	N	N	50.7	17.9
015	43	Figure A9	N	Y	160.7	58.1
016	136	Figure A6	Y	N	11.8	9.7
017	120	Figure A5	Y	N	6.3	14.7
019	54	Figure A7	Y	N	26.4	16.7
021	71	Figure A10	Y	N	165.5	157.5
022	59	Figure A8	N	Y	49.6	29.0
023	93	Figure A10	Y	N	311.7	139.3
024	86	Figure A6	N	N	13.7	16.9
025	138	Figure A7	N	Y	22.5	17.8
026	81	Figure A6	Y	N	11.0	12.3
027	78	Figure A5	Y	N	4.0	12.0
028	73	Figure A8	N	Y	48.8	13.9
029	115	Figure A6	N	N	10.2	11.3
030	118	Figure A9	N	N	78.4	33.2
031	38	Figure A9	Y	N	111.8	96.0
032	76	Figure A7	Y	N	27.6	31.2
033	53	Figure A5	Y	N	18.7	31.9
034	82	Figure A7	N	Y	11.9	7.3
036	72	Figure A8	Y	N	85.4	74.8
037	81	Figure A8	N	N	21.0	13.1
040	81	Figure A7	N	Y	22.5	14.9

The graphs of the medians in Figure A5, Figure A6 and Figure A7 are mainly on the 0-line. This is in part due to the method; median filtering obviously leads to a quantisation of the density axis. However, the α-trimmed mean, intended to mitigate the quantisation issue, does not provide profiles clearly distinct from the zero line either. The figures with participants having higher cough densities (Figure A8, Figure A9 and Figure A10) start to become more distinct with clear peaks in the density in the morning or evening.
